# Alexithymia as a prognostic risk factor for health problems: a brief review of epidemiological studies

**DOI:** 10.1186/1751-0759-6-21

**Published:** 2012-12-17

**Authors:** Masayo Kojima

**Affiliations:** 1 Department of Public Health, Nagoya City University Graduate School of Medical Sciences, 1 Kawasumi, Mizuho-cho, Mizuho-ku, Nagoya, 467-8601, Japan

**Keywords:** Systematic review, Prospective study, Epidemiology

## Abstract

The number of articles on alexithymia has been steadily increasing since the word “alexithymia” was coined in the 1970s to denote a common characteristic that is observed among classic psychosomatic patients in whom therapy was unsuccessful. Alexithymia, a disorder of affect regulation, has been suggested to be broadly associated with various mental and physical health problems. However, most available evidence is based on anecdotal reports or cross-sectional observations. To clarify the predictive value of alexithymia for health problems, a systematic review of prospective studies was conducted. A search of the PubMed database identified 1,507 articles on “alexithymia” that were published by July 31, 2011. Among them, only 7 studies examined the developmental risks of alexithymia for health problems among nonclinical populations and 38 studies examined the prognostic value of alexithymia among clinical populations. Approximately half of the studies reported statistically significant adverse effects, while 5 studies demonstrated favorable effects of alexithymia on health outcomes; four of them were associated with surgical interventions and two involved cancer patients. The studies that showed insignificant results tended to have a small sample size. In conclusion, epidemiological evidence regarding alexithymia as a prognostic risk factor for health problems remains un-established. Even though alexithymia is considered to be an unfavorable characteristic for disease control and health promotion overall, some beneficial aspects are suggested. More prospective studies with sufficient sample sizes and follow-up period, especially those involving life course analyses, are needed to confirm the contribution of alexithymia to health problems.

## Introduction

Alexithymia, a personality construct that reflects difficulties in affective self-regulation [1], was originally noted by psychotherapists as a common characteristic observed among classic psychosomatic patients in whom therapy was unsuccessful [2]. To date, researchers have revealed that alexithymia is broadly associated with various mental and physical health problems. It is now regarded as a key factor affecting treatment responses across the field of medical practice [3].

How is alexithymia associated with the development of health problems and their prognoses? Some of the possible underlying mechanisms have been well documented in previous review articles [1,4,5]. First, alexithymic individuals have impaired emotion-processing and regulating capacities which might induce disruption of homeostasis through alterations of autonomic, endocrine, and immune activities [1]. Second, alexithymic patients have a limited ability to cope adaptively with stressful situations and tend to have unhealthy behaviors, such as poor nutrition, alcohol and drug use, and a sedentary lifestyle [4,5]. Third, alexithymic patients have difficulties in recognizing their own physical and emotional symptoms, which may be linked to a delay or excessive use of medical support, resulting in a poor prognosis. Finally, because of their difficulties communicating their own inner feelings and a poor understanding of other people’s emotions, alexithymic patients find it difficult to build and maintain close relationships with others and to appropriately utilize social supports in order to protect themselves from the potentially pathological influences of stressful events [6].

Thus, theoretically, there is no doubt that alexithymia plays a key role in disease control and health promotion.

### Review of prospective cohort studies on alexithymia

Prospective cohort studies are the best type of observational design applied to examine the etiology and prognosis of health problems [7]. Since Sifneos introduced the concept of alexithymia in 1973 [2], the number of articles regarding alexithymia has been increasing year by year. According to the review by Taylor and Bagby published in 2004 [8], a search of the PsycINFO database revealed more than 1,000 journal articles on alexithymia, while approximately 120 publications on alexithymia had appeared by the mid-80s. However, most of them were anecdotal or cross-sectional data reports. To update the evidence and clarify the predictive value of alexithymia as a health determinant, a brief systematic review of the epidemiological literature was conducted.

### Method of systematic review

The minimum criteria for studies to be included in the current review were as follows: (1) a prospective design with a clinical or nonclinical population sample, (2) evaluating the effects of baseline alexithymia on health-related outcomes, (3) using validated methods to assess alexithymia, and (4) written in English. To capture the broad linkage between alexithymia and health outcomes, the types of treatments and outcomes were not specified in the process of data collection. *P* ≤ 0.05 was considered statistically significant.

A systematic search was conducted using the PubMed database limiting the period from January 1, 1975 to July 31, 2011. A total of 1,507 documents were identified using [“alexithymia” or “alexithymic”] as search terms. By further adding [“prospective” or “prognostic” or “cohort” or “follow”] to the search terms, the search yielded 193 titles. After excluding 19 articles that were written in non-English languages and 8 articles that were published as a review, the remaining 166 articles were inspected manually. Among them, only 7 articles were identified as those reporting the developmental risks of alexithymia for health problems among nonclinical populations, and 38 articles were identified as those reporting the influences of alexithymia on prognosis or results of interventions among hospital-based populations. The reason for exclusion of most studies was the cross-sectional design of data collection (n = 106). Fifteen studies were excluded even though they were prospectively designed because they treated alexithymia as an outcome. Three articles were excluded because they were editorials or commentaries.

Almost all studies included in the current review used the Toronto Alexithymia Scale (TAS) or TAS-20 [9,10] as a tool for evaluating alexithymia. Only two studies used other tools; one study used the Beth Israel Questionnaire and Schalling-Sifneos Personality Scale [11], and the other study used projective personality tests [12,13]; they were included in the current review.

### Major findings from nonclinical population studies

Table 1 shows the summary of studies examining the effects of alexithymia on health outcomes with a prospective design in a nonclinical population. Seven studies prospectively examined the developmental risks of alexithymia for health problems among nonclinical populations [14-20]. Among them, 3 studies demonstrated the statistically significant adverse risk of baseline alexithymia for subsequent health problems [14-16], 3 studies reported no association [18-20], and one reported the beneficial effect of alexithymia on health [17].

**Table 1:** Studies examining the effects of alexithymia on health outcomes with a prospective cohort design in nonclinical population

**Effects of alexithymia**	**N**	**Population**	**Follow up period**	**Outcomes**	**Country**	**Published year**	**Reference no**
Adverse	2321	General population	20 years	Cardiac death	Finland	2010	14
Adverse	2297	General population	5.5 years	All cause morality	Finland	1996	15
Adverse	54	Police officer	2 years	PTSD	USA	2006	16
Beneficial	1207	Urban public transit Operators	7.5 years	Low back pain	USA	2007	17
No association	333	Subsample selected from general population cohort study sample	7 years	Depression	Finland	2010	18
No association	154	General population	30-22 years	Neck-shoulder and low-back pain	Finland	1991	19
No association	43	Fire fighter	2 years	PTSD	Switzerland	2005	20

Two studies were based on the same large cohort data derived from the Finnish general male population and demonstrated the statistically significant adverse effects of alexithymia on total mortality and/or cardiac death [14,15]. A recently conducted cross-sectional study has reported results which may support the findings of these studies. Grabe et al examined 1,168 subjects who were randomly selected from the general population and found a significant association of alexithymia with hypertension and atherosclerotic plaques [21]. The authors pointed out that alexithymia may serve as a long-standing risk factor as well as a familial and genetic factor that deregulates the autonomic nervous system [21]. Autonomic nervous deregulation may positively influence the developmental risk for cardiac death and total mortality.

The most recently published Finnish report failed to show a significant association between baseline alexithymia and subsequent psychiatric diagnoses, such as major depression, personality disorder, and alcohol use disorders, which were confirmed by a structured clinical interview [18]. However, the report is based on the subsample data (n = 290) selected from a general population cohort (n = 2,050) by the presence or absence of chronic high mental symptoms. The study subjects were limited to those who completed the successive 3-year follow-up surveys (1998, 1999, and 2001) and a 7-year follow-up survey including a structured interview (2005). It cannot be denied that a considerable number of alexithymic participants might have developed mental disorders before 2005 and quit the study, which may have caused underestimation of the impact of alexithymia. In addition, the authors included previous depressive symptoms (BDI sum score) in logistic regression analysis to estimate the developmental risk of depression and that might have caused over adjustment [22]. In fact, the results of simple Chi square tests suggested significantly higher prevalence of such psychiatric diagnoses among those who had a TAS-20 sum score above the median [18].

Two studies examined the predictive value of alexithymia on the development of low-back pain (LBP) [17,19]. One study found no association between baseline alexithymia that was evaluated by projective personality tests and the reported severity of neck–shoulder pain and/or LBP among 154 subjects, randomly selected from the general population [19]. The other study examined 1,207 municipal bus drivers and found a favorable association between alexithymia and the 7.5-year incidence of compensated LBP [17]. Inversely, the same research group reported an adverse association between alexithymia and LBP claims from cross-sectional data derived from the same population [23]. The authors speculated that the prevalence of self-reported LBP symptoms and the incidence of compensated LBP claims are not comparable even within the same population, and it might be difficult for alexithymic patients to complete the bureaucratic process to receive compensation, resulting in the low prevalence of compensated LBP [17].

Two studies examined the developmental risks of alexithymia for post-traumatic stress disorder (PTSD) and the results were inconsistent [16,20]. One study examined 43 male firefighters for 2 years and found no significant risk of alexithymia [20]. The other study examined 54 police officers and reported that their TAS-20 scores significantly predicted PTSD symptoms for 2 years [16]. Both studies have too small sample size to estimate the developmental risk of PTSD associated with alexithymia. Further studies with sufficient sample size and follow-up period are necessary to confirm the vulnerability of alexithymic individuals to PTSD.

### Major findings from clinical population studies

A summary of studies examining the effect of alexithymia on health outcomes with a prospective design in a clinical population is shown in Table 2. In total, 38 articles were identified that reported the clinical impacts of alexithymia on prognosis [24-61]. Major types of participants were patients with psychiatric or psychosomatic illness recruited consecutively and the main outcome was the treatment response. Most of them were preliminary, naturalistic studies without control groups. Some studies assigned subjects to a specific treatment or other intervention, but none reported the blinding procedure. Overall, information to evaluate the risk of bias of each study is very limited; therefore, the presence of serious risk of bias should be considered to interpret the results.

**Table 2:** Studies examining the effects of alexithymia on health outcomes with a prospective cohort design in clinicalpopulation

**Type of participants**	**Follow up period**	**Type of treatment**	**Type of main outcome**	**N**	**Country**	**Ref. no.**
Adverse effect						
Psychiatric inPT	4, 8-12W	Multimodal psychotherapy	Global severity Index and depression severity	480	Germany	24
Substance users	10+15W	Motivational intervention	Response to treatment	260	USA	25
Psychiatric outPT	12-21 W	Short-term psychotherapy	Psychiatric symptomatology	251	Canada	26
Hemodialysis outPT	5Y	Hemodialysis therapy	All cause mortality	230	Japan	27
Hemodialysis outPT	6M	Hemodialysis therapy	Depression deterioration	230	Japan	28
OutPT with possible	6Y	Pyschotherapy	Recovery from depression	121	Finland	29
Veterans with military sexual trauma.	7W	Specialized residential treatment	Symptom persistence	175	USA	30
Women taking elective surgical abortion	2M	Surgical abortion	Re-experience and avoidance	140	Netherlands	31
OutPT with functional gastrointestinal disorders	6M	Unspecified treatment	Response to treatment	112	Italy	32
PT taking implantable cardioverter defibrillator	2-5.5 Y	ICD placement	Posttraumatic stress	107	Switzerland	33
Eating disorder PT	3Y	Drug treatment and psychotherapy	PT’ compliance and types of treatments	102	France	34
Eating disorder PT	3Y	Unspecified treatment	Response to treatment	102	France	35
OutPT with major depression	1Y	Unspecified treatment	Response to treatment	86	Finland	36
PT with asthma	2Y	Unspecified treatment	Emargency room visits and QOL (SF-36)	76	Spain	37
OutPT with major depression	10W	Antidepressant	Reduction of depression severity	65	Turkey	38
PT with type 1 Diabetes	8W	Inpatient treatment	Decrease in HbA1c	64	Belgium	39
Alcohol abuser	15W	Inpatient treatment	Maintaining abstinent	46	France	40
PT with somatoform and anxiety disorder	2Y	Inpatient treatment	Symptom persistence	30	Austria	41
Beneficial effect						
Cancer PT	6M	Multicomponent psychological intervention	Pain	104	Italy	42
PT taking in vitro fertilization	6M	In vitro fertilization	Delivery of a living infant	81	Greece	43
Colorectal cancer PT	3Y	Surgery	QOL (SF-36)	60	Italy	44
Ulcerative colotis PT	17M	Pelvic pouch surgery	Psychosocial adjustment	53	Sweden	45
Gynecologic PT	1M	Laparoscopy or laparotomy	QOL (SF-36)	40	Italy	46
No association						
Psychiatric inPT	4, 8-12W	Psychodynamic group therapy	Global severity Index and depression severity	297	Germany	47
OutPT with unexplained physical symptom	6W	Unspecified treatment	Symptom persistence	127	Netherland	48
Pregnant women	1M	Unspecified	Depression development	149	Italy	49
PT with psoriasis	6M	Cognitive behavioral therapy	Response to treatment	80	UK	50
Obese outPT	8M	Behavioral program	Compliance and weight loss	68	Italy	51
InPT taking respiratory rehabilitation	4W	Respiratory rehabilitation	Functional recovery	60	Italy	52
Panic disorder PT	6M	CBT	Response to treatment	55	Switzerland	53
Psychiatric consultation outPT	1Y	Psychotherapy	PT’s compliance	54	Finland	54
OCD inPT	31-139 D	Multimodal CBT	Response to treatment	42	Germany	55
Bulmia nervosa PT	10W	Drug treatment	Symptom improvements	41	England	56
CFS outPT	18M	Unspecified treatment	Symptom improvements	40	Netherland	57
OutPT with psoriasis	3M	Delmatological treatment	Response to treatment	40	France	58
OCD inPT	6Y	Inpatient treatment	OCD deteriotion	34	Switzerland	59
Schizophrenia outPT	1Y	Appropriate treatment	Symptom improvements	29	Italy	60
Eating disorder inPT	1Y	Psychoeducation	Dietary restraint	19	Switzerland	61

PT=patient, OCD=obsessive-compulsive disorders, CFS=chronic fatigue syndrome, CBT=cognitive behavioral therapy, W=week, M=month, Y=year.

The number of studies reporting the adverse effects of alexithymia was 18 [24-41], which was approximately 50% of the total studies. Five studies demonstrated the beneficial effects of alexithymia on clinical outcomes [42-46]. One-third of the identified clinical studies (n = 15) reported no statistically significant associations between baseline alexithymia and treatment outcomes [47-61]. The studies that failed to find significant associations between alexithymia and clinical outcomes tended to have small sample sizes (median = 54, min = 19, max = 297) [47-61] compared with those that demonstrated significant results (median = 103, min = 30, max = 480) [24-46].

The influence of alexithymia on the treatment process and outcomes has been discussed intensively by Lumley et al [4,5]. They speculated that alexithymic patients might respond poorly to psychological treatments, although perhaps not to cognitive behavioral techniques because the compulsive nature and external focus of people with alexithymia may prompt greater adherence to structured exercises and behavioral recommendations [4]. The current review does not identify clear differences by the types of therapy in the influences of alexithymia on the outcomes. However, relatively consistent beneficial effects of alexithymia were observed in studies with surgical interventions [43-47].

To date, the association between surgical treatments and alexithymia has rarely been discussed. Kakatsaki et al examined 81 women undergoing an in vitro fertilization program during a 6 month period and found that alexithymia predicted better outcome; delivery of a living infant [43]. Ripetti et al examined a series of 60 colorectal cancer patients with a three-month follow-up and found that a high-level alexithymia group (TAS-20 ≥51) showed more improvement in QOL measured by SF-36 test after surgery than did the low-level alexithymia group [44]. Weinryb et al examined 53 consecutive patients undergoing pelvic pouch surgery and followed them for 16 to 41 months [45]. They found that the level of alexithymia measured by the Beth Israel Questionnaire was inversely correlated with worse psychological distress and adjustment in relationships at home after surgery. Battista et al examined 40 consecutive patients with benign gynecologic pathology who underwent laparoscopy or laparotomy and found that patients with low-level of alexithymia (TAS-20≤ 59) showed a worsening of QOL score measured by SF-36 after a surgical procedure [46]. In general, surgical treatment procedures are highly structured and active so that the externally oriented thinking style of alexithymic patients may suit them well. Ripetti et al speculated that patients with alexithymia might perceive invasive surgery to be effective because surgical treatment leaves a visible body sign [44]. Weinryb et al suggested that patients with alexithymia may tend to have avoidant coping strategies to the negative aspects of surgery such that they may have better recovery during the postoperative period [45]. On the contrary, a study investigating short term re-experiencing and avoidance after surgical abortion conducted by van Emmerik et al demonstrated a significant adverse effect of alexithymia on the outcomes [31]. Because of the variety of the study backgrounds and the types of treatments and outcomes, we cannot draw definite conclusions from the available data. Further research will be necessary to fully disclose the favorable aspects of alexithymia on treatment outcomes.

One prospective study involving cancer patients conducted by Tulipani showed a significant beneficial effect of alexithymia on reduction in pain perception by psychological intervention to cancer patients [42]. They recruited study participants consecutively, and randomly allocated them to an intervention group or a control group. A total of 52 cancer patients were provided the 6-month psychological intervention with a variety of therapeutic approaches according to guidelines issued in the literature on alexithymia and cancer pain and compared to 52 controls. Psychological intervention significantly reduced alexithymia as well as pain. Multiple regression analysis showed that baseline alexithymia and psychological intervention were both independently associated with a reduction in pain perception. The role of alexithymia in cancer patients is still under study [62]. The effectiveness of intervention focusing on the reduction of alexithymia to improve QOL should be examined among cancer patients and other populations in future research.

Most studies examined the associations between baseline alexithymia and treatment outcomes, whereas only one study reported the 5-year total mortality risk of alexithymia [27]. Interestingly, the increased risk of total mortality associated with alexithymia in hemodialysis patients (multivariate adjusted hazard ratio = 3.62; 95% CI: 1.32–9.93) was higher than that observed among Finnish middle-aged men (1.96; 1.31–2.94) [15]. Whether or not the impact of alexithymia on total mortality may differ by populations should be further investigated.

### Issues for future epidemiological research on alexithymia

Although alexithymia has been considered an unfavorable personality dimension for health promotion and disease prevention, the results of epidemiological studies were inconsistent. Most of them are preliminary and have many methodological problems. Apparently, we need more systematic, prospective studies with sound design to verify each pathway explaining the relationship between alexithymia and health problems that have been theoretically suggested [5]. There are some important points to be followed when conducting an epidemiological study to clarify the influence of alexithymia on health. First, alexithymia is considered a relatively stable characteristic, but it is actually dependent on psychological and/or physical conditions. Therefore, alexithymia should be chronologically measured repeatedly in the same individuals. Second, alexithymia and negative affect are closely associated. Also, interactions with social support cannot be dismissed when examining the influence of alexithymia on health problems. Thus, social support as well as negative affect must be assessed simultaneously when evaluating alexithymia to exclude potential confounding in statistical analysis. Third, if the pre-test score is extremely high, it is likely to show a drop in the post-test score; “regression to the mean [63].” People with alexithymia tend to have higher scores of distress than people without alexithymia. Therefore, simple subtraction of the post-treatment score from the pre-treatment score may cause overestimation of the treatment effect. Using the residual gain score, which is the difference between the actual post-test score and the score that was predicted from a regression equation is one method to adjust the phenomenon, and the use of the analyses of covariance method is also recommended [63]. Finally, as suggested by Grabe et al, alexithymia is considered to be a long-standing risk factor as well as a familial and genetic factor of health [21]. To detect the association between alexithymia and the developmental risk of specific health problems, a long observational period is required. Especially, the association between alexithymia and the onset of depression may occur at a relatively early stage of life. If so, prospective study of the middle-aged population may omit persons who have already developed depression due to alexithymia and overlook the association between them.

### Alexithymia might explain social health inequality

Marmot, who directed the Whitehall Study, a longitudinal epidemiological study of British civil servants, pointed out from his 30 years of research that there are great health inequalities throughout the world that are related to social hierarchy [64-66]. According to Marmot’s speculations, what profoundly affects our health and longevity is not income or lifestyle but autonomy and the opportunities for complete social participation; these follow social gradients and result in health inequalities [66]. Externally oriented thinking styles may inhibit alexithymic patients from feeling like they are in control, and their difficulty in “identifying and describing ones’ own inner feelings” may make them reluctant to participate in social activities. To date, there are two published studies reporting the association between alexithymia and long-term total mortality [15,27], and both of them supported the above hypothesis. Considering its construct, alexithymia might be a key health determinant, as Marmot suggested [65]. In order to resolve social inequalities and improve our health, we should approach the structure of the whole society to reduce alexithymia urgently. Recently, several researchers reported successful interventions to reduce the level of alexithymia [42,51,67,68]. We need more evidence to establish the treatment strategy for alexithymia.

### How to approach alexithymia

The etiology of alexithymia has not been completely determined. Recent studies revealed the contribution of genetic factors to alexithymia development [69-74]. Several studies suggested that the social environment of early life and cultural factors influence alexithymia development [75-77]. Alexithymia is also known to develop secondarily as a reaction to stressful situations [78]. Moreover, some constructs may overlap with alexithymia [4,79], such as emotional intelligence, emotional awareness, empathy deficits [80,81], and autism spectrum disorders [82,83]. How a person develops alexithymic characteristics and how it affects his or her health throughout the life course need to be clarified. We also need to know how to approach alexithymic patients when their developmental backgrounds are varied.

Current review has revealed some favorable influences of alexithymia on surgical treatment outcomes [42-46]. A positive influence of alexithymia on behavioral treatments and adherence to treatment recommendations has been observed in several previous studies [4]. Lumley et al speculated that the compulsive nature and external focus of alexithymic patients prompt greater adherence to structured exercises and behavioral recommendations [4]. If clinicians and family members understand the characteristics of alexithymic patients and provide them with appropriate support, they might exhibit good compliance and thereby achieve good health outcomes. This point should be further explored in future studies.

### Study limitation

To conduct the current systematic review, only the PubMed database for a limited period was used for the article search. The results of unpublished studies or articles not registered for PubMed were not included. Therefore, this systematic review did not cover all existing studies regarding alexithymia and the data need to be updated.

Almost all epidemiological studies reviewed in this article used the TAS or its short version, the TAS-20, to evaluate alexithymia. Both of them are self-measure questionnaires and have been used widely because of their good reliability and feasibility [84]. However, it has been argued that people with severe alexithymia may not evaluate their symptoms correctly because of having difficulty perceiving their inner feelings [85]. Moreover, a significant positive correlation of the TAS or the TAS-20 score and negative affect has been reported consistently [84]. The original authors of the TAS have recommended the measurement of alexithymia with several tools using different methods [8]. Whether or not we should use multiple measures of alexithymia has been very well discussed in the review article by Lumley et al [4]. Even though the application of interview-based assessment to epidemiological studies with large samples is difficult, the differences in measuring tools, especially those that include objective measures, should be verified in the future.

## Conclusions

The epidemiological evidence regarding alexithymia as a prognostic risk factor for health problems is insufficient. Prospective studies with sufficient sample sizes will be necessary in order to confirm the contribution of alexithymia to health problems.

## Competing interests

The author declares that she has no competing interests.
